# Innate function of house dust mite allergens: robust enzymatic degradation of extracellular matrix at elevated pH

**DOI:** 10.1186/s40413-017-0154-3

**Published:** 2017-07-04

**Authors:** Kumiko Oida, Lukas Einhorn, Ina Herrmann, Lucia Panakova, Yvonne Resch, Susanne Vrtala, Gerlinde Hofstetter, Akane Tanaka, Hiroshi Matsuda, Erika Jensen-Jarolim

**Affiliations:** 1 The interuniversity Messerli Research Institute of the University of Veterinary Medicine Vienna, Medical University Vienna and University Vienna, Veterinaerplatz 1, 1210 Vienna, Austria; 2 Cooperative Major in Advanced Health Science, Graduate School of Bio-Applications and System Engineering, Tokyo University of Agriculture and Technology, Saiwai-cho 3-8-5, Fuchu, Tokyo 183-8509 Japan; 3 Department for Companion Animals and Horses, University of Veterinary Medicine Vienna, Veterinaerplatz 1, 1210 Vienna, Austria; 4 Institute of Pathophysiology and Allergy Research, Center for Pathophysiology, Infectiology and Immunology, Medical University Vienna, Waehringer Guertel 18-20, 1090 Vienna, Austria; 5 Christian Doppler Laboratory for the Development of Allergen Chips, Medical University Vienna, Waehringer Guertel 18-20, 1090 Vienna, Austria; 6 Laboratory of Comparative Animal Medicine Division of Animal Life Science, Institute of Agriculture, Tokyo University of Agriculture and Technology Division of Animal Life Science, Institute of Agriculture, Saiwai-cho 3-8-5, Fuchu, Tokyo 183-8509 Japan; 7 Laboratory of Veterinary Molecular Pathology and Therapeutics, Division of Animal Life Science, Institute of Agriculture, Tokyo University of Agriculture and Technology, Saiwai-cho 3-8-5, Fuchu, Tokyo 183-8509 Japan

**Keywords:** *Dermatophagoides pteronyssinus*, Allergens, Epithelial barrier, Extracellular matrix, Proteases, *N*-acetyl-β-hexosaminidase

## Abstract

**Background:**

Exposure to the house dust mite *Dermatophagoides pteronyssinus* (D.p.) increases the risk for developing allergic diseases in humans and their best friends, the dogs. Here, we explored whether this allergenic mite via its enzymes may impact the cutaneous extracellular matrix (ECM), which critically determines epithelial barrier integrity both structurally and functionally.

**Methods:**

Two extracts obtained from either dust-purified or cultured D.p. bodies were used in the present study. To assess the potential impact of D.p. on protein components of the ECM, proteolytic activity of the D.p. extracts were determined by casein and gelatin gel zymography, and their *N*-acetyl-β-hexosaminidase activity determined colorimetrically. In addition, IgE-dependent and innate degranulation potential of D.p. was examined in canine MPT-1 mast cells and neurite outgrowth assay using rat pheochromocytoma PC-12 cells.

**Results:**

In gel zymography, both extracts digested the substrates casein and gelatin in a dose-dependent manner, especially at alkaline pH, and effective in a wide range of temperatures (30 °C−42 °C). In particular, a 25-kDa band corresponding to Der p 1, the major D.p. allergen for humans, was found enzymatically active in both casein and gelatin gels regardless of the presence of metal ions and of alkaline conditions. Besides protease activity, *N*-acetyl-β-hexosaminidase activity was detected in both extracts, suggesting that D.p. affects the cutaneous ECM through deteriorating both proteins and glycosaminoglycans. While both D.p. extracts induced IgE-dependent mast cell degranulation, much less innate effects on mast- and neuronal cells were observed.

**Conclusions:**

Our data highlight that D.p. is a robust source of several distinct enzymes with protease- and *N*-acetyl-β-hexosaminidase activities. In alkaline milieu they can degrade components of the ECM. Therefore, D.p. may contribute to epithelial barrier disruption especially when the skin surface pH is elevated.

**Electronic supplementary material:**

The online version of this article (doi:10.1186/s40413-017-0154-3) contains supplementary material, which is available to authorized users.

## Background

*Dermatophagoides pteronyssinus* (D.p.) represents one of the most frequently encountered house dust mite (HDM) species worldwide [1]. Exposure to D.p. allergens raises the risk of developing allergic diseases, such as asthma [2] or atopic dermatitis (AD) [3]. Especially, those allergens categorized as group 1 (Der p 1 in D.p.; Der f 1 in *Dermatophagoides farinae*, D.f.), potently induce allergic responses, and also break tight junctions between airway epithelial cells by cysteine protease activity, and thereby alter the permeability [4]. In accordance, papain, a structural homolog of group 1 allergens, affects tight junction permeability of the *epidermis* [5], and activates mast cells (MCs) [6].

This seems important as epithelial barrier dysfunction, and even more -disruption, is accepted as the initial event before cutaneous sensitization occurs [7]. In line with the “outside-inside-outside” concept a defect in tissue-related genes, as the example of loss-of-function Filaggrin alleles in AD [8], was proposed to support hypersensitivity. In addition to the genetic predisposition, environmental factors play a crucial role and recent studies indicate that a rise in skin surface pH might be a major determinant for a barrier defect [9, 10]. The enzymatic activity of Der p 1 as outlined above is an example of an environmental factor; however, dogs, which share common immune principles [11] and milieu with humans [12], are less likely to react to group 1 allergens, in spite of the fact that HDMs are a major source of environmental allergens for dogs as well as for humans [13]. Accordingly, the mechanisms by which D.p. frequently cause hypersensitivity in humans and animals, seem more than the action of a single enzyme.

The cutaneous extracellular matrix (ECM) consists mainly of proteins associated with glycosaminoglycans (GAGs) and glycoproteins. The architecture and composition of these components determines the biophysical properties of the skin, such as stiffness, compliance and resilience [14]. The basement membrane, representing a thin but firm meshwork of collagen and laminin, underlies the basal layer of the epidermis and thereby shields the host from the outside environment [15]. The body’s own matrix metalloproteases (MMPs) are responsible for the tissue remodeling and ECM degradation. However, several invading pathogens produce ECM-degrading enzymes or may alter host-derived proteolytic activity [16].

In addition to structural defects, ECM breakdown leads to an alternation in behavior of multiple cell types in the skin. Collagen fragmentation, which is frequently associated with aged [17] or photo damaged skin [18], results in dysfunction of keratinocytes or fibroblasts. Cleavage products of hyaluronan (HA), known as a major GAG in ECM, serve as signals of tissue injury, which enhance inflammatory cytokine production and differentially modulate function of macrophages and dendritic cells through pattern recognition receptors [19]. Therefore, exposure to foreign enzymes with potential for ECM deterioration is a likely cause of epidermal barrier defects.

In this study, we aimed to determine in vitro whether and under which conditions, D.p. allergens have the potency to affect the cutaneous ECM and thereby initiate skin barrier disruption. Further, to verify whether such potency is compatible with allergenic activity, we examined the innate effect of D.p. allergens on MCs ex vivo, and the specific effect using canine serum obtained from dogs with canine atopic dermatitis (CAD) which is clinically and pathologically similar to the human disease [20].

## Methods

### Extracts from mite bodies

The whole mite bodies: the purified D.p. from dust samples 4966 (Allergon, Aengelholm, Sweden) for extract I; the cultured D.p. LG-8444 (Cosmo Bio LSL, Tokyo, Japan) for extract II, were homogenized in sterile phosphate-buffered saline with 0.1 mM PMSF, a serine protease inhibitor. This supernatant was collected by centrifugation. Proteins were quantified by the method of Bradford.

### Reagents

Polyacrylamide gels were prepared using 30% acrylamide solution (AppliChem, Darmstadt, Germany). In zymography, gels were copolymerized casein (C3400) or gelatin (G2500) (both from Sigma-Aldrich, St. Luis, MO) as a protein substrate. In a colorimetric measurement of *N*-acetyl-β-hexosaminidase (β-HEX) activity, its chromogenic substrate 4-nitrophenyl *N*-acetyl-β-D-glucosaminide (NP-GlcNAc) (Sigma-Aldrich) was applied. For the induction of MC degranulation, dog IgE P115 was purchased from Bethyl Laboratories (Montgomery, TX), anti-dog anti-IgE antibody SM1498P from Acris Antibodies (Herford, Germany), and Ca-ionophore A23187 from Sigma-Aldrich. For Western blot analysis, horseradish peroxidase-conjugated goat anti-dog anti-IgE antibody NB7346 was purchased from Novus Biologicals (Littleton, CO). In neurite outgrowth bioassay, recombinant mouse β-nerve growth factor (NGF) was purchased from R&D Systems (Minneapolis, MN). Unless otherwise indicated, all chemicals used in this study were obtained from Sigma-Aldrich.

### Zymography

D.p. extracts or recombinants were diluted with 125 mM Tris-HCl (pH 6.8) and the following 2 × sample buffer (20% glycerol, 4% sodium dodecyl sulfate: SDS, 125 mM Tris-HCl, 0.01% bromophenol blue, pH 6.8) with neither 2-mercaptoethanol nor boiling. Samples were separated at 4 °C in 10% SDS-polyacrylamide gel electrophoresis (SDS-PAGE) copolymerized with 0.1% casein or gelatin. Migrated enzymes were renatured from SDS by soaking in 2.5% Triton X-100 for 30 min twice, and then activated in 50 mM Tris-HCl, pH 8.0, for casein; 50 mM Tris-HCl, 5 mM CaCl_2_, 1 mM ZnCl_2_, pH 7.6, for gelatin, at 37 °C for 16 h. To chelate metal ions, after separation and after SDS removal, gelatin gels were incubated in the reaction buffer supplemented with 50 mM EDTA for Ca^2+^ or 10 mM 1,10-phenanthroline (Phen) for Zn^2+^. The optimal pH and temperature were evaluated by changing the buffer conditions during digestion. Thermal susceptibility was determined by heating extracts (10 μg) prior to an assay. Gels were stained with 0.2% Coomassie Brilliant Blue (CBB) R-250 solution. Digestion bands were analyzed using the Java-based image processing program ImageJ (The National Institutes of Health, Bethesda, MD).

### Recombinant allergens

The recombinant D.p. allergens used in our experiments are described in Additional file 1: Table S1. In brief, allergen proteins were expressed in *Escherichia coli* and purified to homogeneity. The purified proteins were analysed by SDS-PAGE with CBB or silver staining. For SDS-PAGE, recombinants were diluted with 2 × sample buffer containing 10% 2-mercaptoethanol, and then boiled at 95 °C for 7 min. Zymography was performed as mentioned above. A specification of the recombinants is shown in Additional file 1: Table S1.

### Measurement of β-HEX activity

To determine β-HEX activity, various concentrations of D.p. extracts (0.1−50 μg/ml) or recombinants (25 μg/ml) were incubated at 37 °C for 1 h with 3 mM NP-GlcNAc in 100 mM citrate buffer, pH 4.5. The enzymatic reaction was stopped by 200 mM glycine, pH 10.7. The accumulation of degrading products was measured by the absorbance at 405 nm using the Infinite M200 PRO plate reader (TECAN, Maennedorf, Switzerland). Thermal lability was determined by heating extracts (50 μg/ml) prior to a colorimetric assay and the relative β-HEX activity was calculated as a ratio of the optical density (OD) value without heating. All experiments were performed in duplicate.

### Canine sera

Sera were obtained from 26 canine patients diagnosed with atopic dermatitis (CAD according to ICADA guidelines [21]), in the Dermatology Department of the University of Veterinary Medicine Vienna (Vienna, Austria). In line with the routine diagnostic procedures, all atopic dogs underwent an intradermal skin test. The total IgE levels and the serological reactivity to D.p. were assessed in all specimens. Those that specifically reacted to D.p. allergens were used in this study (Additional file 1: Table S2).

### Western blot analysis

Recombinant allergens were diluted with 2 × reducing sample buffer and then boiled at 95 °C for 7 min. Samples, 168.8 ng/lane for recombinant (r) Der p 15; 143.8 ng/lane for rDer p 18, were separated in 10% SDS-PAGE and transferred onto PVDF membrane (Millipore, Bedford, MA). The membrane was blocked by 1% bovine serum albumin/Tris-buffered saline, 0.05% Tween-20 and incubated overnight at 4 °C with diluted canine sera (the final IgE concentration: 30–100 ng/ml). Allergen-specific IgE bonds were detected with horseradish peroxidase-labeled anti-IgE.

### MC degranulation assay

MC degranulation assay was performed by measurement of β-HEX release in the canine MC line MPT-1, which harbors the high-affinity IgE receptor FcεRI, essentially as described [22], using anti-IgE (5 μg/ml) or A23187 (5–50 μM). Cells were cultured in α-MEM with 10% fetal bovine serum (FBS) (both from Life Technologies, Carlsbad, CA) and antibiotics. Cells were preloaded with serum IgE from canine patients allergic to D.p., at 37 °C for 2 h. The optimal IgE concentration had been determined by titrating each serum in pre-experiments to 5–10 μg/ml IgE. Then preloaded cells were challenged (37 °C, 1 h) with D.p. extracts (50 μg/ml). Following incubation, cell-free conditioned medium was examined for β-HEX content using NP-GlcNAc as mentioned earlier. To eliminate the direct effect of extracts on the substrate, the OD values obtained from those cells which were incubated at 4 °C with D.p. extracts (50 μg/ml), were subtracted. All experiments were performed in triplicate. The degranulation rate was expressed as present of total content by the following formula: degranulation rate (%) = (OD_supernatant_/(OD_supernatant_ + OD_lysate_)) × 100.

### Neurite outgrowth bioassay

Neurite outgrowth experiments were performed in the rat pheochromocytoma PC-12 (Japanese Collection of Research Bioresources Cell Bank, Osaka, Japan) using NGF (50 ng/ml). Cells were maintained in RPMI1640 (Life Technologies) with 5% FBS, 10% horse serum (Thermo Fisher Scientific, Waltham, MA) and antibiotics. In bioassays, following NGF priming for 7 days, cells were treated with D.p. extracts (100 μg/ml) for 48 h in a collagen-coated dish. In control experiments, cells were incubated with or without NGF. A percentage of neurite-bearing cells, which extended neurites longer than their cell body, was calculated from random 5 fields under microscopy.

### Statistics

Statistical examinations were performed using SigmaPlot® 11 (Systat Software, San Jose, CA). All data represent the mean ± standard error of the mean (SEM). *p* value of < 0.05 was considered statistically significance in difference (^*^, *p* < 0.05; ^**^, *p* < 0.01; ^***^, *p* < 0.001).

## Results

### Protease activity in D.p. extracts

**Fig. 1:**
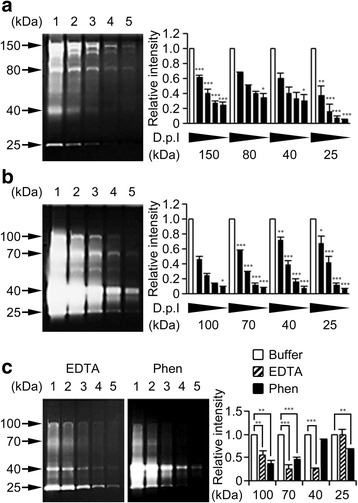
D.p. extracts exhibit proteolytic activity. **a** Casein and **b** gelatin zymography of extract I (D.p. I). Intensity of arrow-indicated bands was normalized by those detected in the first lane (10 μg, *white columns*) and evaluated by one-way analysis of variance (ANOVA) with Tukey tests. **c** Gelatin zymography of D.p.I using EDTA and Phen. Intensity of arrow-indicated bands in the first lane (10 μg) was normalized by those detected without chelators (*white columns*) and difference in data with or without chelators were compared by Student’s t-tests. Photos, *gray scale* images of the representative data. Error bars, ± SEM (*n* = 3). The total protein load per gel lane: the lane 1, 10 μg; 2, 5 μg; 3, 2.5 μg; 4, 1 μg; 5, 0.5 μg

The proteolytic activity repertoire of D.p. was examined by gel zymography. This technology is based on non-reducing SDS-PAGE copolymerized substrate proteins (i.e., casein or gelatin). Following electrophoretic separation of the enzyme of interest, protease activity can be visualized by CBB staining as a digestion band at the molecular weight (MW) of the enzyme. To evaluate the potential impact of any contaminating commensal factors, 2 different D.p. extracts of distinct origin were analyzed: D.p. extract I from dust-purified mites, and extract II from cultured mites. In fact SDS-PAGE depicted slight differences in their protein composition (Additional file 1: Figure S1). Zymography showed dose-dependent protein digestion, albeit with distinct patterns of MW (Fig. 1a, b and Additional file 1: Figure S2a, b). These digestion bands suggest the existence of multiple proteases in the extract. To distinguish one from another, the biochemical properties of extracts were analyzed.

MMPs belong to a family of Ca^2+^-dependent, Zn^2+^-containing endopeptidases [23] whose homologs are found in genomes of both vertebrates and invertebrates [24]. Some of these enzymes potently lyse gelatin; to clarify their involvement in gelatinolysis, catalytic dependencies on the metal ions were determined by use of chelators: EDTA, for Ca^2+^; Phen, for Zn^2+^. In D.p. extract I, the 70- and 100-kDa bands were markedly inhibited by both agents (Fig. 1c). The digestion of the 40-kDa band was partially diminished by EDTA, but not affected by Phen (Fig. 1c). Similarly, gelatinolysis with high MW detected in extract II were effectively inhibited by the chelators (Additional file 1: Figure S2c). In contrast, the approximately 25-kDa band, which was detected in casein and gelatin gels using both extracts, was significantly suppressed by Phen, but not by EDTA (Fig. 1c and Additional file 1: Figure S2c).

### Optimal conditions for D.p. protease activity

**Fig. 2:**
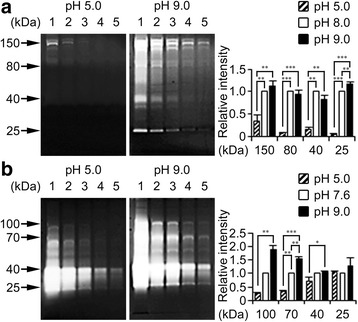
D.p. proteases are optimally active at elevated pH. **a** Casein and **b** gelatin zymography of extract I (D.p.I) digested at pH 5.0 or pH9.0. Intensity of arrow-indicated bands in the first lane (10 μg) was normalized by those detected at pH 8.0 (casein) or pH 7.6 (gelatin, both in *white columns*). Statistical evaluation was performed by one-way ANOVA with Tukey tests. Photos represent *gray scale images* of the typical data. Error bars, ± SEM (*n* = 3). The total protein load per gel lane: the lane 1, 10 μg; 2, 5 μg; 3, 2.5 μg; 4, 1 μg; 5, 0.5 μg

**Fig. 3:**
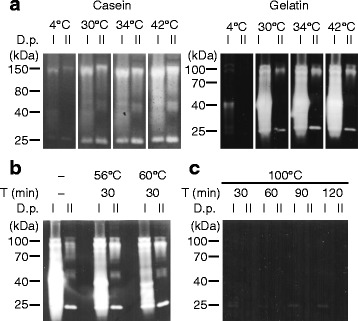
D.p. proteases are active at a wide range of temperatures. **a** Casein and gelatin zymography digested at the indicated temperatures. **b** Gelatin zymography of both extracts which were preheated at the indicated temperatures before being electrophoretically separated. **c** Gelatin zymography for both extracts after boiling them at 100 °C for different time ranges. Photos represent *gray scale images* of the representative data. T, time; D.p.I, extract I; D.p.II, extract II. In each experiment, the total protein load per gel lane was 10 μg

The optimal pH and temperature for the catalytic activity were determined with a change of buffer conditions. After electrophoretic separation and after SDS removal, gels were incubated 37 °C at pH 5.0 or pH 9.0. In both extracts, casein digestion hardly occurred at pH 5.0, while it was dramatically enhanced at pH 9.0 (Fig. 2a and Additional file 1: Figure S3a). Although gelatin digestion remained predominant at pH 5.0 towards low MW compounds of extract I, elevation in pH again intensified protease activity (Fig. 2b and Additional file 1: Figure S3b). These results, together with data in Fig. 1 and Additional file 1: Figure S2, indicate that most of these proteases are activated optimally at neutral to alkaline pH (i.e., pH 8.0, for casein; pH 7.6, for gelatin). On the other hand, protein digestion capacity of the D.p. extracts was only prevented by incubation at 4 °C, but operative over a wide range of temperatures from 30 °C to 42 °C (Fig. 3a).

### Protease activity of recombinant allergens

The thermal susceptibility and tolerance of D.p. protease activity was evaluated by heating the extracts, prior to enzymatic analysis, at a series of temperatures from 40 °C to 60 °C for 30 min, which are common temperatures for washing clothes. Heating at 56 °C for 30 min had seemingly no effects on gelatin digestion by both extracts (Fig. 3b), neither 40 °C, 45 °C nor 50 °C (data not shown). Heating at 60 °C for 30 min only partially diminished gel digestion (Fig. 3b). However, when the temperature reached 100 °C, neither of extracts degraded gelatin gels at a detectable level (Fig. 3c). Overall, there were no marked differences in heat susceptibility between the proteases.

**Fig. 4:**
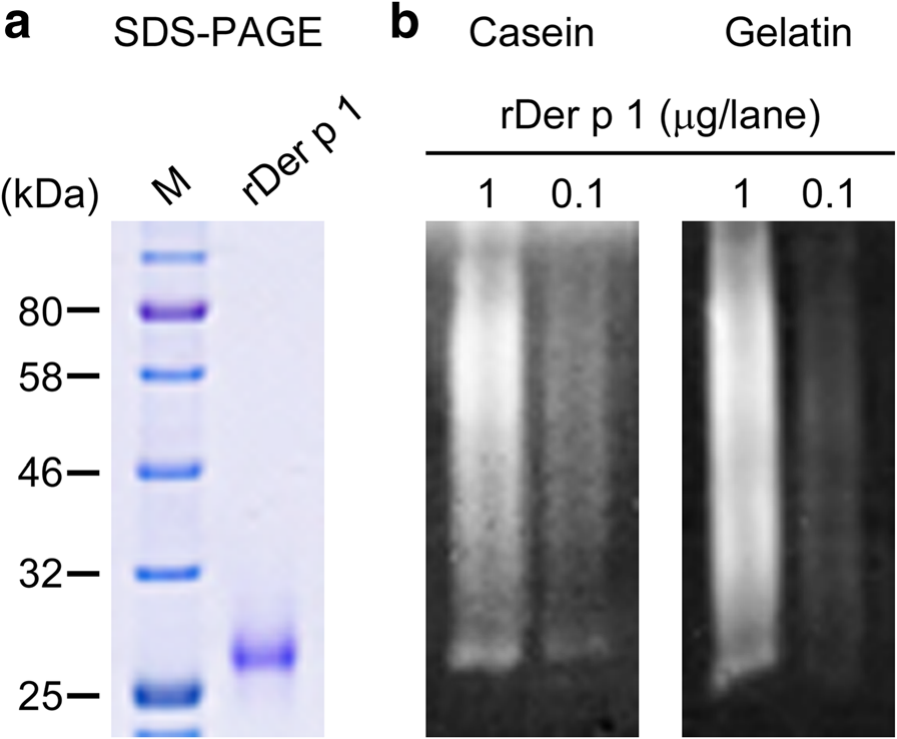
Recombinant Der p 1 allergen degrades casein and gelatin gels. **a** SDS-PAGE with CBB staining of rDer p 1. The total load per gel lane was 1 μg. **b** Casein and gelatin zymography using the indicated amounts of rDer p 1. Photos represent *gray scale images* of the representative data. M, molecular marker

Der p 1 is a 25-kDa allergen that is a homolog to cysteine proteases [25]. Its conformational epitopes are readily denatured at 75 °C for 1 h or 100 °C for 10 min [26]. It has been also reported that this allergen is irreversibly denatured in acidic pH (pH 2.0) but resistant to mild alkaline conditions (pH 10.0) [26]. Accordingly, Der p 1 is supposed to be responsible for the digestion band appearing at 25 kDa, and purified rDer p 1 was applied to verify its activity upon the substrates. SDS-PAGE showed a protein migrating at approximately 25 kDa under reducing conditions (Fig. 4a). Zymography showed robust degradation by rDer p 1 on both gels, albeit with seemingly different mobility, most likely due to dimerization of recombinants (Fig. 4b). However, the other allergens within the range of recombinants available to us for this study (Additional file 1: Table S1) had no effect on casein and gelatin gels (Additional file 1: Figure S4a, b).

### β-HEX activity in D.p. extracts

**Fig. 5:**
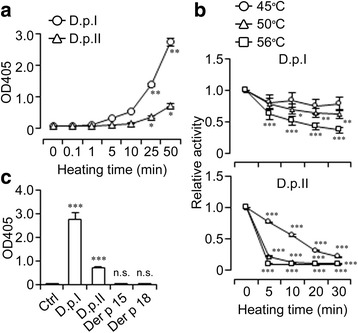
D.p. extracts exhibit β-HEX activity. **a** β-HEX activity in the indicated concentrations of both extracts. An increase in OD values was statistically evaluated by one-way ANOVA with Dunnett’s tests. **b** Heat lability of β-HEX found in both extracts (50 μg/ml). The relative activity was calculated as OD_with heating_/OD_without heating_ and evaluated by two-way ANOVA with Holm-Sidack tests. **c** β-HEX activity in extracts (50 μg/ml) or rDer p 15 and 18 allergens (25 μg/ml). Significance in an increased OD was determined by Student’s t-tests. Error bars, ± SEM (*n* = 3); n.s., no significance in difference; D.p.I, extract I; D.p.II, extract II; Ctrl, buffer alone

The hydrolytic activity towards GAG components is of specific relevance for chitinase allergens, and was examined with focus on β-HEX (the Enzyme Commission number: EC 3.2.1.52), now including *N*-acetyl-β-glucosaminidase (EC 3.2.1.30). This enzyme is known as a lysosomal enzyme that hydrolyzes glycosidic linkages between *N*-acetyl-β-glucosamine (GlcNAc) and glucuronate. Hence, β-HEX activity in D.p. extracts was colorimetrically measured using its chromogenic substrate NP-GlcNAc. Both extracts induced a dose-dependent increase in the OD at 405 nm, indicating accumulation of the degradation products (Fig. 5a). β-HEX activity in D.p. extracts appeared susceptible to heating at temperatures over 50 °C for D.p. extract I; over 45 °C for extract II (Fig. 5b). All experiments were conducted at pH 4.5, indicating that β-HEX enzymatically functions under acidic conditions.

**Fig. 6:**
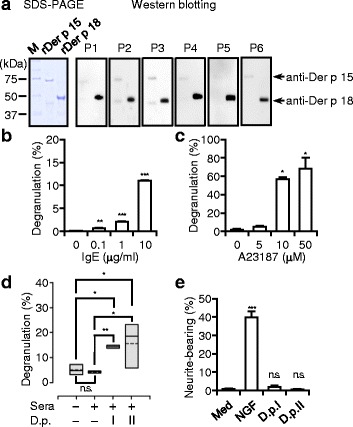
D.p. extracts have effects on MCs though the IgE-FcεRI axis. **a** SDS-PAGE of recombinants (300 ng) and Western blotting (168.8 ng, for rDer p 15; 143.8 ng of rDer p 18 per gel lane) of specific IgE in canine sera. **b** MC degranulation by IgE cross-linkage, by **c** A23187, or **d** canine sera and extracts. Results were evaluated by one-way ANOVA with **b** Holm-Sidack, **c** Dunnett’s test, and **d** Student’s *t*-test. *Boxes*, the distribution of results (*n* = 3); *solid* and *broken lines*, the median and the mean. **e** Neurite outgrowth bioassay. A comparison with medium alone (*Med*) was performed by Student’s t-tests. Error bars, ± SEM (*n* = 3); n.s., no significance in difference; D.p,I, extract I; D.p.II, extract II

Chitinase (EC 3.2.1.14) hydrolyzes glycosidic bonds between GlcNAcs of chitin, known as the most abundant GAG found in arthropod exoskeleton. Interestingly, IgE reacts to group 15 (chitinases) and 18 (chitinase-like proteins) of mite allergens at high frequencies in dogs [27] (Fig. 6a), but not in humans [28]. However, when rDer p 15 or rDer p 18 were applied to the colorimetric assays, neither caused a significant increase in OD values (Fig. 5c), contradicting the involvement of exactly these mite chitinases in NP-GlcNAc hydrolysis.

### Effects of D.p. extracts on MCs

The main manifestations of allergic skin, such as eczema or erythema, strongly depend on activation of MCs or basophils [29]. Thus, allergenic activity of D.p. was determined by the effect of D.p. extracts on MCs. Sera from pet dogs suffering from CAD containing D.p.-specific IgE, as revealed by testing on blotted canine major allergens Der p 15 and Der p 18. Twenty four sera of 26 tested samples reacted via IgE to one or both allergens (data not shown); taking into account specific IgE levels and skin test positivity to D.p., 6 serum samples were selected for further analysis (Fig. 6a and Additional file 1: Table S2). Degranulation in canine MPT-1 cells could be dose-dependently triggered by canine IgE-FcεRI crosslinking (Fig. 6b) comparable with that induced by human antibodies [22], and also by Ca-ionophore A23187 (Fig. 6c). Hence, MPT-1 cells were adapted to a novel ex vivo bioassay using the canine sera. As shown in Fig. 6d, mediator release from cells preloaded with canine D.p.-IgE could be triggered by both D.p. extracts, but none of the extracts had any effect in the absence of specific IgE (data not shown).

Intractable pruritus in AD is resistant to conventional treatments such as antihistamines, suggesting contributing factors other than MC mediators. We hypothesized that the increased nerve density histologically found in AD lesions might be responsible for the persistent itch sensation [30]. Therefore, any potential actions of D.p. extracts on nerve elongation were determined by neurite outgrowth bioassay using PC-12 cells. While NGF in approximately 40% of cells induced neurite outgrowth (Fig. 4e), neither of the D.p. extracts had any effect on the PC-12 cells (Fig. 4e and Additional file 1: Figure S5).

## Discussion

The present study demonstrates that D.p. has a potent enzymatic impact on the ECM. This may lead to epithelial barrier defects, thereby explaining the high prevalence of cutaneous hypersensitivity to D.p. in humans and animals.

By zymography casein and gelatin digestion capacity could be demonstrated in extracts obtained from both dust-purified or cultured mites. This method has long been used for studies of ECM-degrading enzymes particularly of MMPs with gelatin, the denatured form of collagen [23]. Further, casein is favorably applied to readout of a wide range of serine and cysteine proteases, such as kallikreins [9], in the absence of metal ions. Accordingly, the in vitro gels using purified proteins (i.e., casein and gelatin) represent an experimental approximation to the in vivo situations. Gelatin digestion at high molecular mass bands in the gels which was diminished by both chelators, might be attributed in part to enzymes classified into the MMP family. In contrast, the chelators had much less effect on digestion of bands at a low molecular mass. These findings indicate that D.p. extracts contain several different kinds of enzymes that can degrade protein components of the cutaneous ECM.

The D.p. proteases were optimally active at neutral to alkaline pH. Therefore, the acidic mantle of healthy skin (i.e., at pH 4.5−5.5 in humans) may not only prevent the overgrowth of indigenous pathobionts [31], but also prevent the activation of D.p. proteases. A rise in skin surface pH has been related to skin defects, such as AD [32]. Further, a more recent study indicates that alkalization of skin surface induces abnormal activation of endogenous serine proteases and thereby disrupts the skin barrier function, even before the onset of AD [10]. The temperature of the skin surface is generally lower than the core body at 37 °C, thereby controlling surface-attached pathogens such as *Staphylococcus aureus* [33]; the fact that casein and gelatin digestion by the D.p. proteases occurred at temperatures lower than 37 °C suggests that they are active at the skin surface. Collectively, these findings raise the possibility that the enzymatic activity of D.p. proteases could be enhanced when the pH of the host skin surface is elevated leading to barrier damage.

This is the first demonstration, to our knowledge, that D.p. extracts exhibit β-HEX activity. β-HEX acts on glucoside- and galactoside- types of GAGs, and functionally overlaps with hyaluronidases, known as HA catabolic enzymes [34], thereby acting as spreading factor in some venom [35]. Most notably, although almost all D.p. allergens are proteins, D.p. extracts induced degranulation of MCs in an IgE-dependent manner, indicating that they possess allergenic potency in parallel to the enzymatic functions. This seems crucially important, given that defected epithelial barrier facilitates secondary sensitization to allergens [7]. In contrast with our results concerning the innate effects on MCs, it has been reported that papain activates human cord blood-derived MCs IgE-independently via protease-activated receptor-2 and its enzymatic activity [6]. This contradiction may be due to low abundance of Der p 1 in the extracts or decreased sensitivity of the canine cells. Collectively, although further investigations are needed, these data support the hypothesis that D.p. may enzymatically inflict damage on protein and also GAG components of the ECM, and subsequently induce allergic responses through an IgE-mediated mechanism.

Finally, commensal organisms of D.p., being determined by various environmental and climatic conditions of mite habitats, may contaminate dust samples and mite cultures. In particular, *Aspergillus* species, known as ubiquitous saprophytic fungi, are favorable food to D.p. [36], which often generate enzymes to degrade mammalian tissue for nutrient acquisition and invasion [37]. Therefore, to this end we cannot entirely exclude the possible involvement of such microbial symbionts in the observed enzymatic functions, which may explain the remarkable differences between extract I and II from two different mite breed sources, especially in gelatinolysis under acidic pH, and in the thermal susceptibility of β-HEX.

## Conclusions

The present study highlights the crucial role of D.p. carrying a variety of enzymes with a potential for degrading ECM and acting as allergens. Given that activation of a large proportion of D.p. proteases is abrogated by acidic pH of skin surface, elevated skin pH can be a critical host factor required for really developing disease following D.p. exposure. This novel finding highlights that skin pH regulation is an important strategy for prevention and treatment in this type of dermatitis. In conclusion, our findings demonstrate that enzymatic potency of D.p. in alkaline conditions damages the cutaneous ECM and therefore weakens the host defense in humans and their best friend, the dog.

## Additional file

Additional file 1: Appendix. Methods. **Figure S1.** Distinct band pattern of proteins in D.p. extracts. **Figure S2.** Proteolytic activity of D.p. extract II. **Figure S3.** The pH optimum of proteolysis by D.p extract II. **Figure S4.** Proteolytic activity of recombinant D.p. allergens. **Figure S5.** Innate effects of D.p. extracts on neurite outgrowth. **Table S1.** Specification of recombinant D.p. allergens. **Table S2.** Specification of canine subjects with CAD. (PPTX 13.0 mb)

## Abbreviations

AD: Atopic dermatitis
ANOVA: Analysis of variance
CAD: Canine atopic dermatitis
CBB: Coomassie Brilliant Blue
D.f.: *Dermatophagoides farinae*
D.p.: *Dermatophagoides pteronyssinus*
EC: Enzyme Commission number
ECM: Extracellular matrix
FBS: Fetal bovine serum
FcεRI: High-affinity IgE receptor
GAG: Glycosaminoglycan
GlcNAc: *N*-acetyl-β-glucosamine
HA: Hyaluronan
HDM: House dust mite
β-HEX: *N*-acetyl-β-hexosaminidase
MC: Mast cell
MMP: Matrix metalloproteinase
MW: Molecular weight
NGF: Nerve growth factor
NP-GlcNAc: 4-nitrophenyl *N*-acetyl-β-D-glucosaminide
OD: Optical density
Phen: 1,10-phenanthroline
r: Recombinant
SDS: Sodium dodecyl sulfate
SDS-PAGE: SDS-polyacrylamide gel electrophoresis
SEM: Standard error of the mean

## References

[1] Arlian LG, Morgan MS, Neal JS (2002). Dust mite allergens: ecology and distribution. Curr Allergy Asthma Rep.

[2] Gent JF, Belanger K, Triche EW, Bracken MB, Beckett WS, Leaderer BP (2009). Association of pediatric asthma severity with exposure to common household dust allergens. Environ Res.

[3] Teplitsky V, Mumcuoglu KY, Babai I, Dalal I, Cohen R, Tanay A (2008). House dust mites on skin, clothes, and bedding of atopic dermatitis patients. Int J Dermatol.

[4] Wan H, Winton HL, Soeller C, Tovey ER, Gruenert DC, Thompson PJ (1999). Der p 1 facilitates transepithelial allergen delivery by disruption of tight junctions. J Clin Invest.

[5] Stremnitzer C, Manzano-Szalai K, Willensdorfer A, Starkl P, Pieper M, Konig P (2015). Papain degrades tight junction proteins of human keratinocytes in vitro and sensitizes C57BL/6 mice via the skin independent of its enzymatic activity or TLR4 activation. J Invest Dermatol.

[6] Seaf M, Ben-Zimra M, Mankuta D, Dayan N, Levi-Schaffer F (2016). Papain activates human mast cells to release proinflammatory mediators via its enzymatic activity. J Invest Dermatol.

[7] Bieber T (2008). Atopic dermatitis. N Engl J Med.

[8] Palmer CN, Irvine AD, Terron-Kwiatkowski A, Zhao Y, Liao H, Lee SP (2006). Common loss-of-function variants of the epidermal barrier protein filaggrin are a major predisposing factor for atopic dermatitis. Nat Genet.

[9] Deraison C, Bonnart C, Lopez F, Besson C, Robinson R, Jayakumar A (2007). LEKTI fragments specifically inhibit KLK5, KLK7, and KLK14 and control desquamation through a pH-dependent interaction. Mol Biol Cell.

[10] Jang H, Matsuda A, Jung K, Karasawa K, Matsuda K, Oida K (2015). Skin pH is the master switch of kallikrein 5-mediated skin barrier destruction in a murine atopic dermatitis model. J Invest Dermatol.

[11] Jensen-Jarolim E, Einhorn L, Herrmann I, Thalhammer JG, Panakova L. Pollen allergies in humans and their dogs, cats and horses: differences and similarities. Clin Transl Allergy. 2015. doi:10.1186/s13601-015-0059-6. 10.1186/s13601-015-0059-6 PMC4387677 25852853

[12] Marsella R, Girolomoni G (2009). Canine models of atopic dermatitis: a useful tool with untapped potential. J Invest Dermatol.

[13] Nuttall TJ, Hill PB, Bensignor E, Willemse T (2006). House dust and forage mite allergens and their role in human and canine atopic dermatitis. Vet Dermatol.

[14] Krieg T, Aumailley M (2011). The extracellular matrix of the dermis: flexible structures with dynamic functions. Exp Dermatol.

[15] Rowe RG, Weiss SJ (2008). Breaching the basement membrane: who, when and how?. Trends Cell Biol.

[16] Steukers L, Glorieux S, Vandekerckhove AP, Favoreel HW, Nauwynck HJ (2012). Diverse microbial interactions with the basement membrane barrier. Trends Microbiol.

[17] Brun C, Jean-Louis F, Oddos T, Bagot M, Bensussan A, Michel L (2016). Phenotypic and functional changes in dermal primary fibroblasts isolated from intrinsically aged human skin. Exp Dermatol.

[18] Quan T, Little E, Quan H, Qin Z, Voorhees JJ, Fisher GJ (2013). Elevated matrix metalloproteinases and collagen fragmentation in photodamaged human skin: impact of altered extracellular matrix microenvironment on dermal fibroblast function. J Invest Dermatol.

[19] Muto J, Morioka Y, Yamasaki K, Kim M, Garcia A, Carlin AF (2014). Hyaluronan digestion controls DC migration from the skin. J Clin Invest.

[20] Marsella R, Olivry T, Carlotti DN (2011). Current evidence of skin barrier dysfunction in human and canine atopic dermatitis. Vet Dermatol.

[21] Hensel P, Santoro D, Favrot C, Hill P, Griffin C. Canine atopic dermatitis: detailed guidelines for diagnosis and allergen identification. BMC Vet Res. 2015. doi:10.1186/s12917-015-0515-5. 10.1186/s12917-015-0515-5 PMC4531508 26260508

[22] Amagai Y, Tanaka A, Ohmori K, Matsuda H (2008). Establishment of a novel high-affinity IgE receptor-positive canine mast cell line with wild-type *c-kit* receptors. Biochem Biophys Res Commun.

[23] Snoek-Van Beurden PA, Von Den Hoff JW (2005). Zymographic techniques for the analysis of matrix metalloproteinases and their inhibitors. Biotechniques.

[24] Page-Mccaw A (2008). Remodeling the model organism: matrix metalloproteinase functions in invertebrates. Semin Cell Dev Biol.

[25] Chua KY, Stewart GA, Thomas WR, Simpson RJ, Dilworth RJ, Plozza TM (1988). Sequence analysis of cDNA coding for a major house dust mite allergen, Der p 1. Homology with cysteine proteases. J Exp Med.

[26] Lombardero M, Heymann PW, Platts-Mills TA, Fox JW, Chapman MD (1990). Conformational stability of B cell epitopes on group I and group II *dermatophagoides spp.* Allergens. Effect of thermal and chemical denaturation on the binding of murine IgG and human IgE antibodies. J Immunol.

[27] Mueller RS, Janda J, Jensen-Jarolim E, Rhyner C, Marti E (2016). Allergens in veterinary medicine. Allergy.

[28] Hales BJ, Elliot CE, Chai LY, Pearce LJ, Tipayanon T, Hazell L (2013). Quantitation of IgE binding to the chitinase and chitinase-like house dust mite allergens Der p 15 and Der p 18 compared to the major and mid-range allergens. Int Arch Allergy Immunol.

[29] Tanaka A, Amagai Y, Oida K, Matsuda H (2012). Recent findings in mouse models for human atopic dermatitis. Exp Anim.

[30] Tominaga M, Takamori K (2014). Itch and nerve fibers with special reference to atopic dermatitis: therapeutic implications. J Dermatol.

[31] Ali SM, Yosipovitch G (2013). Skin pH: from basic science to basic skin care. Acta Derm Venereol.

[32] Knor T, Meholjic-Fetahovic A, Mehmedagic A (2011). Stratum corneum hydration and skin surface pH in patients with atopic dermatitis. Acta Dermatovenerol Croat.

[33] Krishna S, Miller LS (2012). Host-pathogen interactions between the skin and *Staphylococcus aureus*. Curr Opin Microbiol.

[34] Gushulak L, Hemming R, Martin D, Seyrantepe V, Pshezhetsky A, Triggs-Raine B (2012). Hyaluronidase 1 and β-hexosaminidase have redundant functions in hyaluronan and chondroitin sulfate degradation. J Biol Chem.

[35] Da Silveira RB, Chaim OM, Mangili OC, Gremski W, Dietrich CP, Nader HB (2007). Hyaluronidases in *Loxosceles intermedia* (Brown spider) venom are endo-β-*N*-acetyl-D-hexosaminidases hydrolases. Toxicon.

[36] Nadchatram M (2005). House dust mites, our intimate associates. Trop Biomed.

[37] Budak SO, Zhou M, Brouwer C, Wiebenga A, Benoit I, Di Falco M et al. A genomic survey of proteases in Aspergilli. BMC Genom. 2014. doi:10.1186/1471-2164-15-523. 10.1186/1471-2164-15-523 PMC4102723 24965873

