# Supplementary material for: Innate function of house dust mite allergens: robust enzymatic degradation of extracellular matrix at elevated pH
Source: World Allergy Organ J. 2017 Jul 4;10(1):23. doi: 10.1186/s40413-017-0154-3 (PMC5496134; doi:10.1186/s40413-017-0154-3)
Supplement: Additional file 1: — Appendix. Methods. Figure S1. Distinct band pattern of proteins in D.p. extracts. Figure S2. Proteolytic activity of D.p. extract II. Figure S3. The pH optimum of proteolysis by D.p extract II. Figure S4. Proteolytic activity of recombinant D.p. allergens. Figure S5. Innate effects of D.p. extracts on neurite outgrowth. Table S1. Specification of recombinant D.p. allergens. Table S2. Specification of canine subjects with CAD. (PPTX 13.0 mb) [file 40413_2017_154_MOESM1_ESM.pptx]

## Slide 1
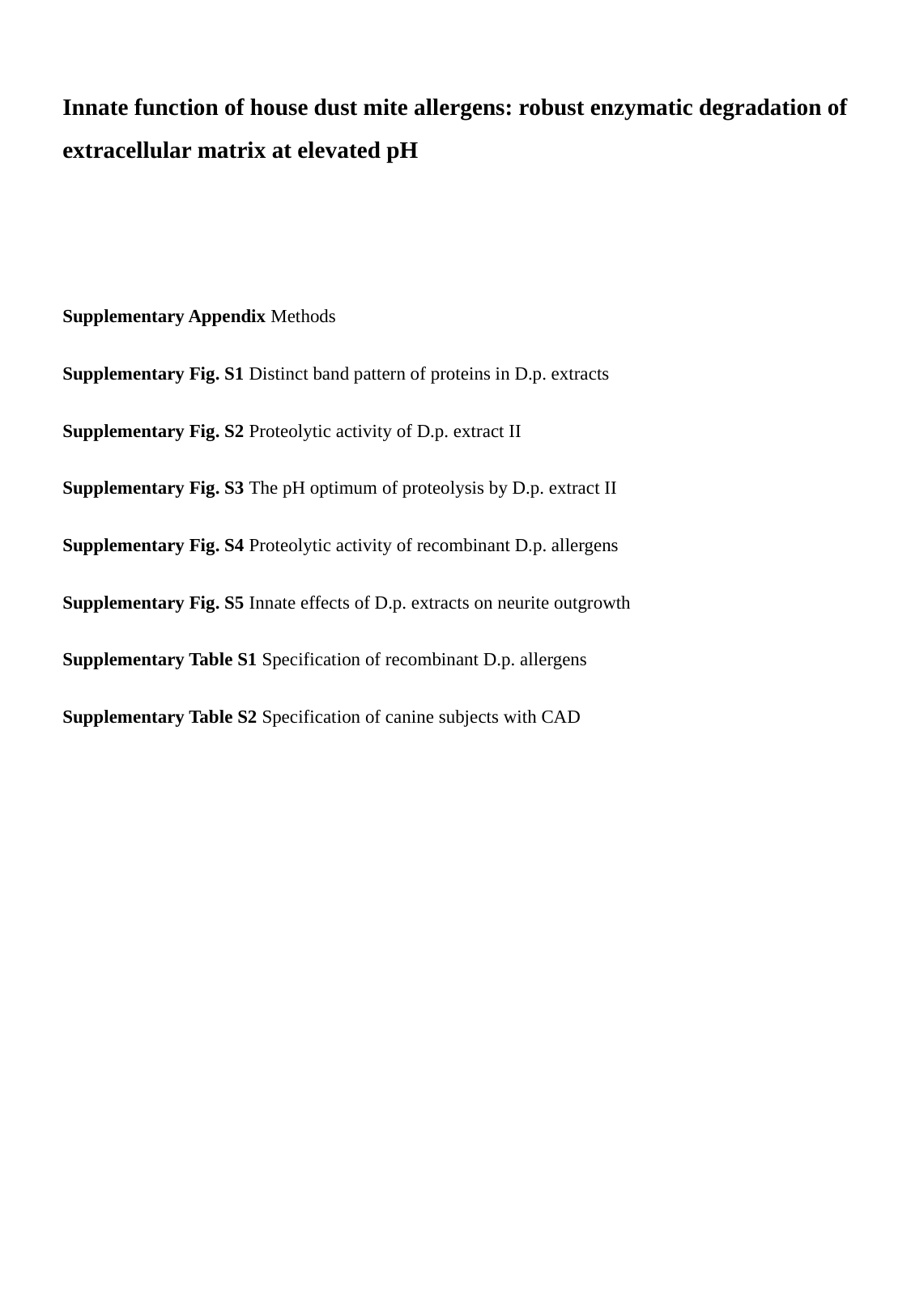

Innate function of house dust mite allergens: robust enzymatic degradation of extracellular matrix at elevated pH
Supplementary Appendix Methods
Supplementary Fig. S1 Distinct band pattern of proteins in D.p. extracts
Supplementary Fig. S2 Proteolytic activity of D.p. extract II
Supplementary Fig. S3 The pH optimum of proteolysis by D.p. extract II
Supplementary Fig. S4 Proteolytic activity of recombinant D.p. allergens
Supplementary Fig. S5 Innate effects of D.p. extracts on neurite outgrowth
Supplementary Table S1 Specification of recombinant D.p. allergens
Supplementary Table S2 Specification of canine subjects with CAD

## Slide 2
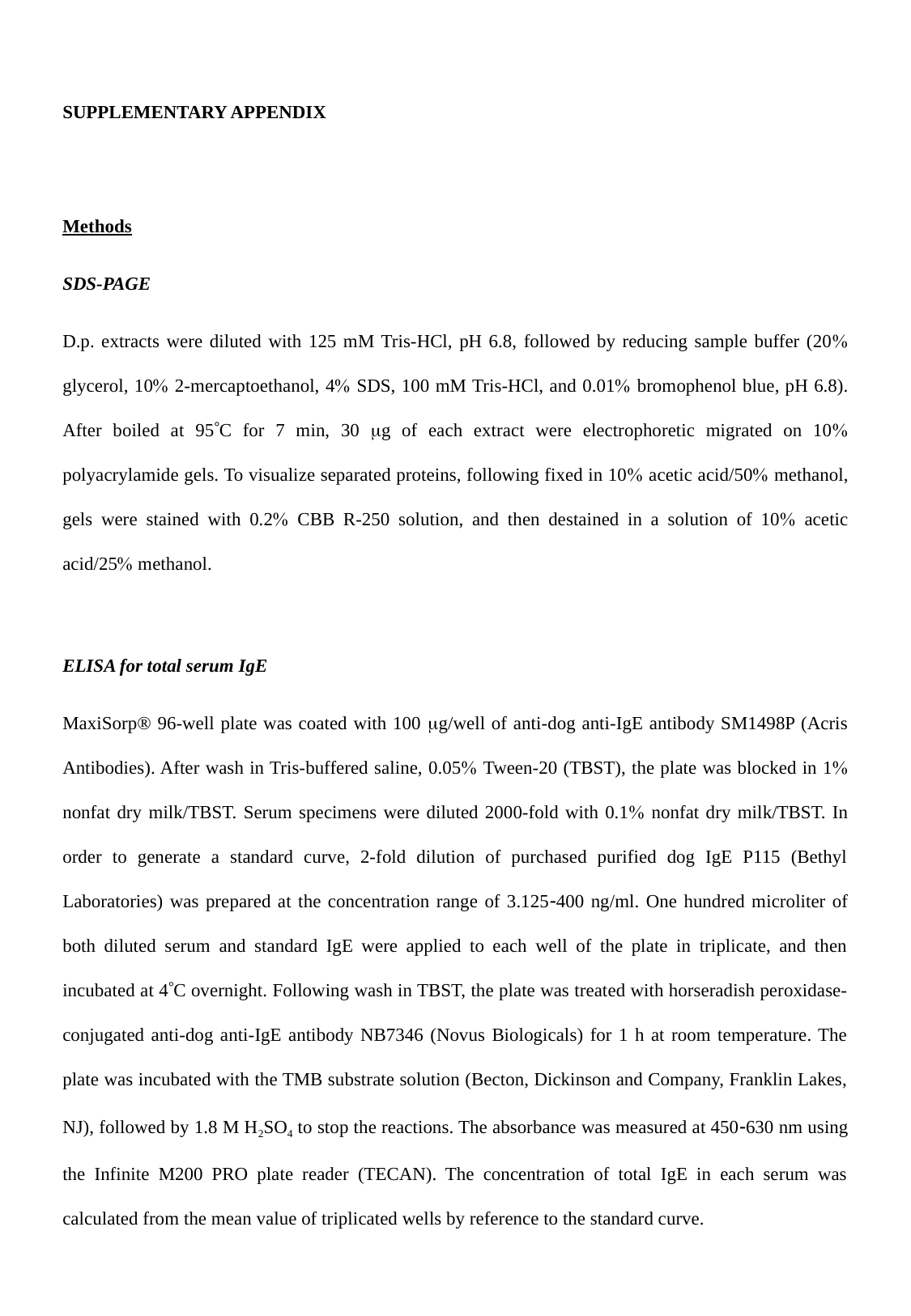

SUPPLEMENTARY APPENDIX
Methods
SDS-PAGE
D.p. extracts were diluted with 125 mM Tris-HCl, pH 6.8, followed by reducing sample buffer (20 glycerol, 10 2-mercaptoethanol, 4 SDS, 100 mM Tris-HCl, and 0.01 bromophenol blue, pH 6.8). After boiled at 95°C for 7 min, 30 μg of each extract were electrophoretic migrated on 10 polyacrylamide gels. To visualize separated proteins, following fixed in 10 acetic acid/50 methanol, gels were stained with 0.2 CBB R-250 solution, and then destained in a solution of 10 acetic acid/25 methanol.
ELISA for total serum IgE
MaxiSorp® 96-well plate was coated with 100 μg/well of anti-dog anti-IgE antibody SM1498P (Acris Antibodies). After wash in Tris-buffered saline, 0.05 Tween-20 (TBST), the plate was blocked in 1 nonfat dry milk/TBST. Serum specimens were diluted 2000-fold with 0.1 nonfat dry milk/TBST. In order to generate a standard curve, 2-fold dilution of purchased purified dog IgE P115 (Bethyl Laboratories) was prepared at the concentration range of 3.125−400 ng/ml. One hundred microliter of both diluted serum and standard IgE were applied to each well of the plate in triplicate, and then incubated at 4°C overnight. Following wash in TBST, the plate was treated with horseradish peroxidase-conjugated anti-dog anti-IgE antibody NB7346 (Novus Biologicals) for 1 h at room temperature. The plate was incubated with the TMB substrate solution (Becton, Dickinson and Company, Franklin Lakes, NJ), followed by 1.8 M H2SO4 to stop the reactions. The absorbance was measured at 450−630 nm using the Infinite M200 PRO plate reader (TECAN). The concentration of total IgE in each serum was calculated from the mean value of triplicated wells by reference to the standard curve.

## Slide 3
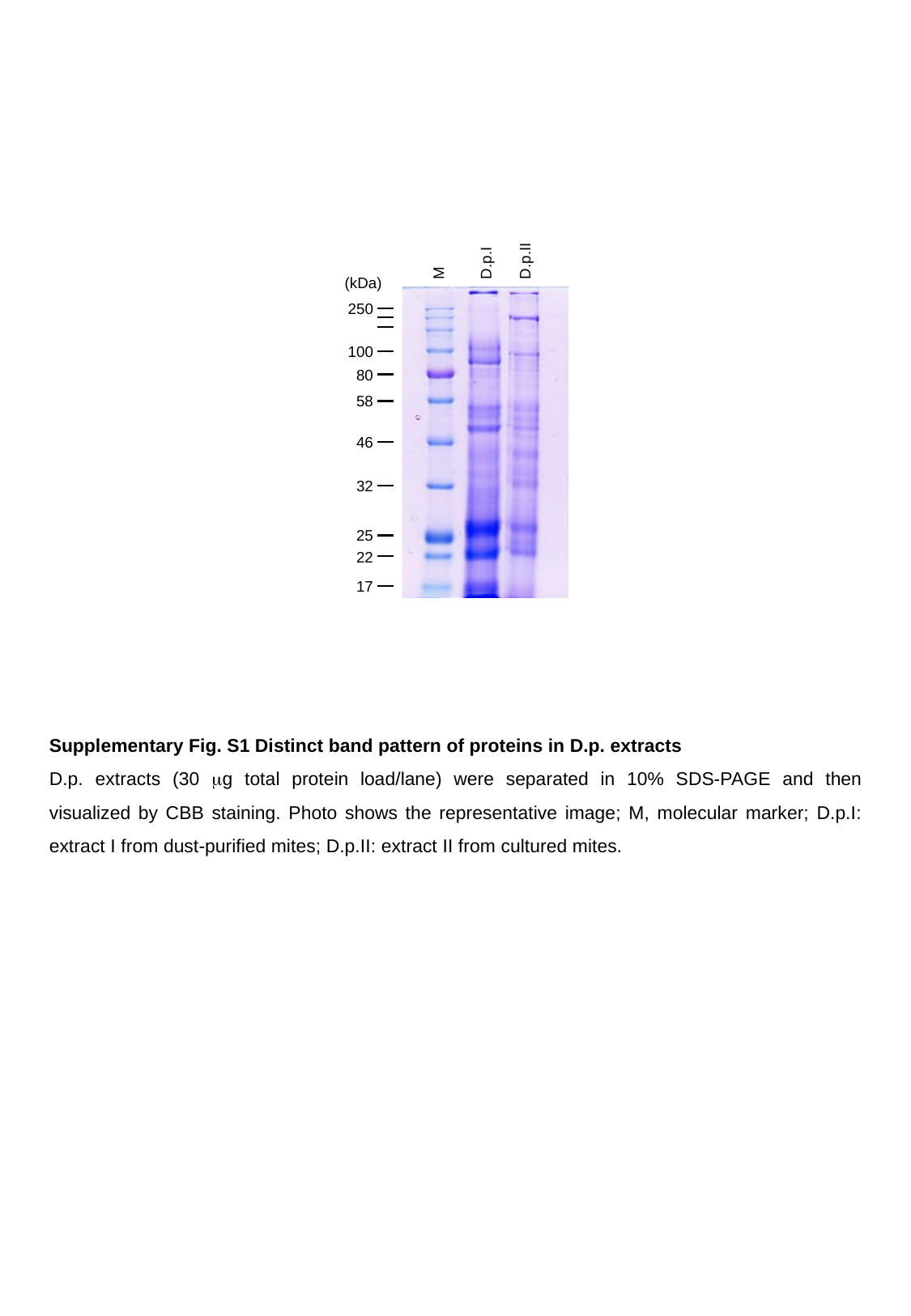

M
D.p.I
D.p.II
(kDa)
250
100
80
58
46
32
25
22
17
Supplementary Fig. S1 Distinct band pattern of proteins in D.p. extracts
D.p. extracts (30 μg total protein load/lane) were separated in 10% SDS-PAGE and then visualized by CBB staining. Photo shows the representative image; M, molecular marker; D.p.I: extract I from dust-purified mites; D.p.II: extract II from cultured mites.

## Slide 4
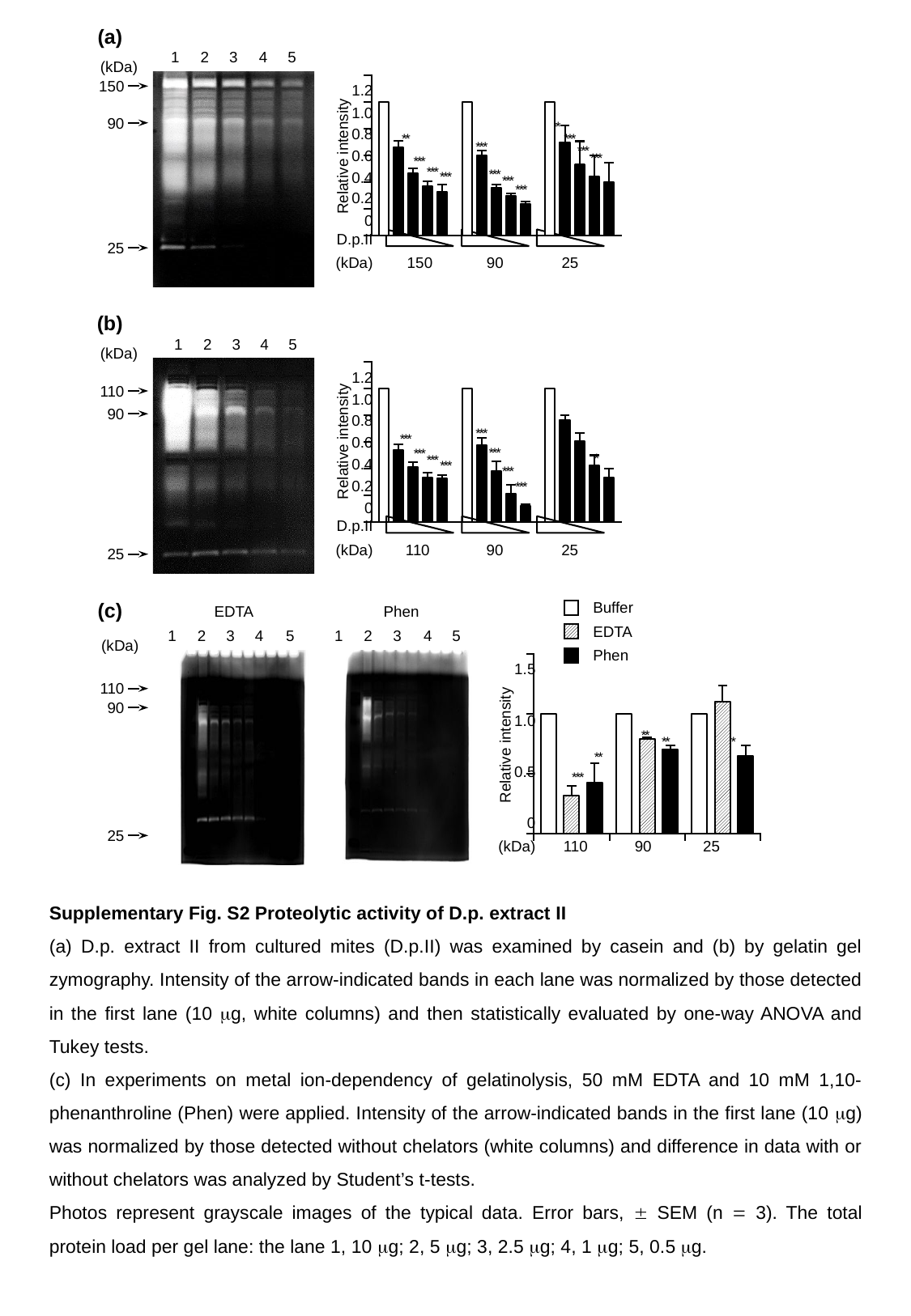

(a)
1
2
3
4
5
(kDa)
150
1.2
1.0
0.8
0.6
Relative intensity
0.4
0.2
0
D.p.II
150
90
25
90
**
**
***
***
***
***
***
***
***
***
***
***
25
(kDa)
(b)
(kDa)
1.2
1.0
0.8
Relative intensity
0.6
0.4
0.2
0
D.p.II
110
90
110
90
25
25
1
2
3
4
5
***
***
***
***
*
***
***
***
***
(kDa)
(c)
EDTA
Phen
(kDa)
110
90
25
1.5
1.0
Relative intensity
0.5
0
110
90
25
1
2
3
4
5
1
2
3
4
5
(kDa)
Buffer
EDTA
Phen
**
**
*
**
***
### Chart
| Category | | | | | |
|---|---|---|---|---|---|
### Chart
| Category | | | | | |
|---|---|---|---|---|---|
### Chart
| Category | | | |
|---|---|---|---|Supplementary Fig. S2 Proteolytic activity of D.p. extract II
(a) D.p. extract II from cultured mites (D.p.II) was examined by casein and (b) by gelatin gel zymography. Intensity of the arrow-indicated bands in each lane was normalized by those detected in the first lane (10 μg, white columns) and then statistically evaluated by one-way ANOVA and Tukey tests.
(c) In experiments on metal ion-dependency of gelatinolysis, 50 mM EDTA and 10 mM 1,10-phenanthroline (Phen) were applied. Intensity of the arrow-indicated bands in the first lane (10 μg) was normalized by those detected without chelators (white columns) and difference in data with or without chelators was analyzed by Student’s t-tests.
Photos represent grayscale images of the typical data. Error bars, ± SEM (n = 3). The total protein load per gel lane: the lane 1, 10 μg; 2, 5 μg; 3, 2.5 μg; 4, 1 μg; 5, 0.5 μg.

## Slide 5
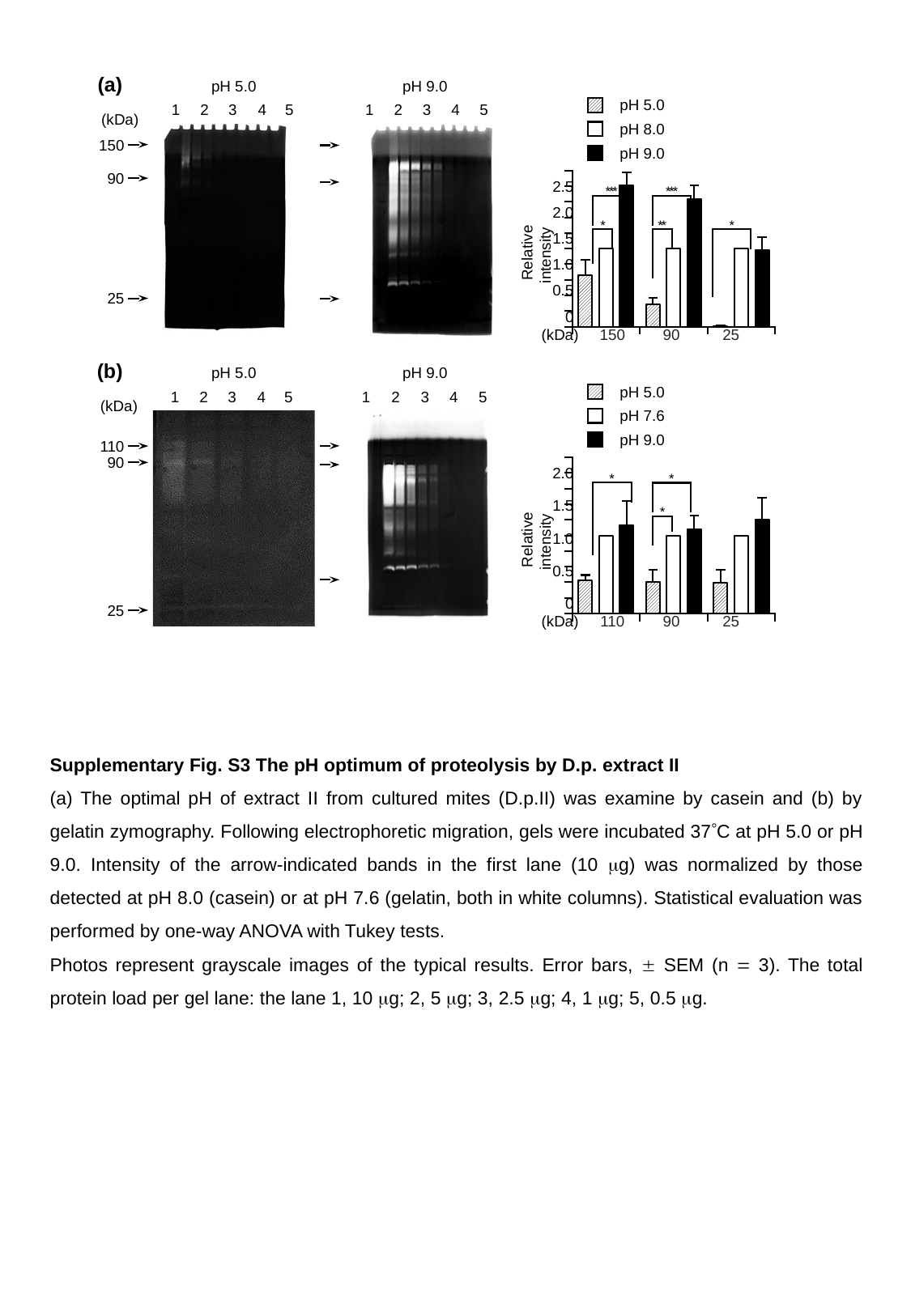

(a)
pH 5.0
pH 9.0
(kDa)
150
90
2.5
2.0
1.5
Relative intensity
1.0
0.5
25
0
150
90
25
pH 5.0
pH 8.0
pH 9.0
***
***
*
**
*
(kDa)
1
2
3
4
5
1
2
3
4
5
(b)
pH 5.0
pH 9.0
(kDa)
110
90
2.0
1.5
1.0
Relative intensity
0.5
0
25
110
90
25
pH 5.0
pH 7.6
pH 9.0
1
2
3
4
5
1
2
3
4
5
*
*
*
(kDa)
### Chart
| Category | | | |
|---|---|---|---|
### Chart
| Category | | | |
|---|---|---|---|Supplementary Fig. S3 The pH optimum of proteolysis by D.p. extract II
(a) The optimal pH of extract II from cultured mites (D.p.II) was examine by casein and (b) by gelatin zymography. Following electrophoretic migration, gels were incubated 37°C at pH 5.0 or pH 9.0. Intensity of the arrow-indicated bands in the first lane (10 μg) was normalized by those detected at pH 8.0 (casein) or at pH 7.6 (gelatin, both in white columns). Statistical evaluation was performed by one-way ANOVA with Tukey tests.
Photos represent grayscale images of the typical results. Error bars, ± SEM (n = 3). The total protein load per gel lane: the lane 1, 10 μg; 2, 5 μg; 3, 2.5 μg; 4, 1 μg; 5, 0.5 μg.

## Slide 6
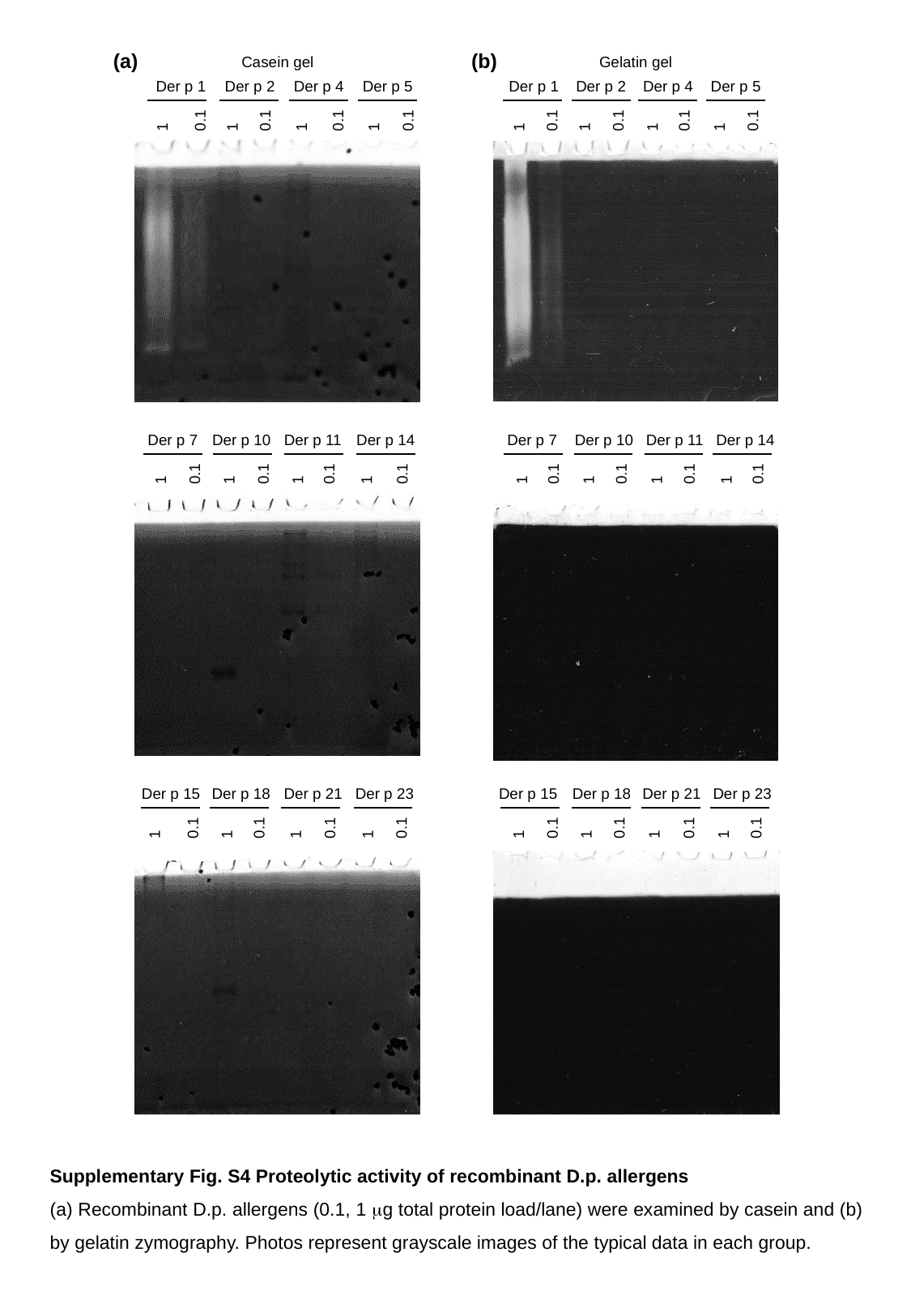

(a)
Casein gel
Der p 1
Der p 2
Der p 4
Der p 5
1
0.1
1
0.1
1
0.1
1
0.1
(b)
Gelatin gel
Der p 1
Der p 2
Der p 4
Der p 5
1
0.1
1
0.1
1
0.1
1
0.1
Der p 7
Der p 10
Der p 11
Der p 14
1
0.1
1
0.1
1
0.1
1
0.1
Der p 15
Der p 18
Der p 21
Der p 23
1
0.1
1
0.1
1
0.1
1
0.1
Der p 7
Der p 10
Der p 11
Der p 14
1
0.1
1
0.1
1
0.1
1
0.1
Der p 15
Der p 18
Der p 21
Der p 23
1
0.1
1
0.1
1
0.1
1
0.1
Supplementary Fig. S4 Proteolytic activity of recombinant D.p. allergens
(a) Recombinant D.p. allergens (0.1, 1 μg total protein load/lane) were examined by casein and (b) by gelatin zymography. Photos represent grayscale images of the typical data in each group.

## Slide 7
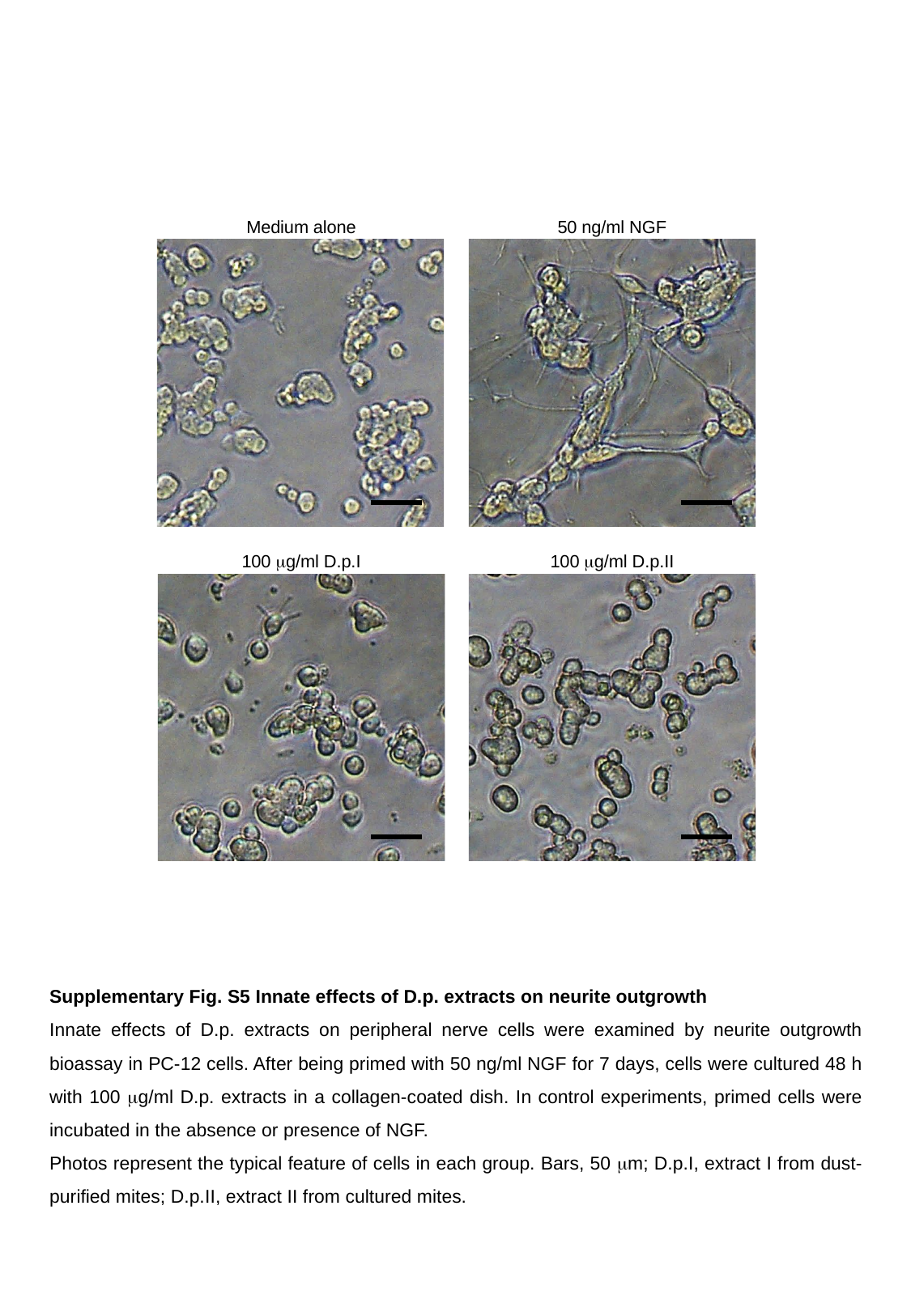

Medium alone
50 ng/ml NGF
100 μg/ml D.p.I
100 μg/ml D.p.II
Supplementary Fig. S5 Innate effects of D.p. extracts on neurite outgrowth
Innate effects of D.p. extracts on peripheral nerve cells were examined by neurite outgrowth bioassay in PC-12 cells. After being primed with 50 ng/ml NGF for 7 days, cells were cultured 48 h with 100 μg/ml D.p. extracts in a collagen-coated dish. In control experiments, primed cells were incubated in the absence or presence of NGF.
Photos represent the typical feature of cells in each group. Bars, 50 μm; D.p.I, extract I from dust-purified mites; D.p.II, extract II from cultured mites.

## Slide 8
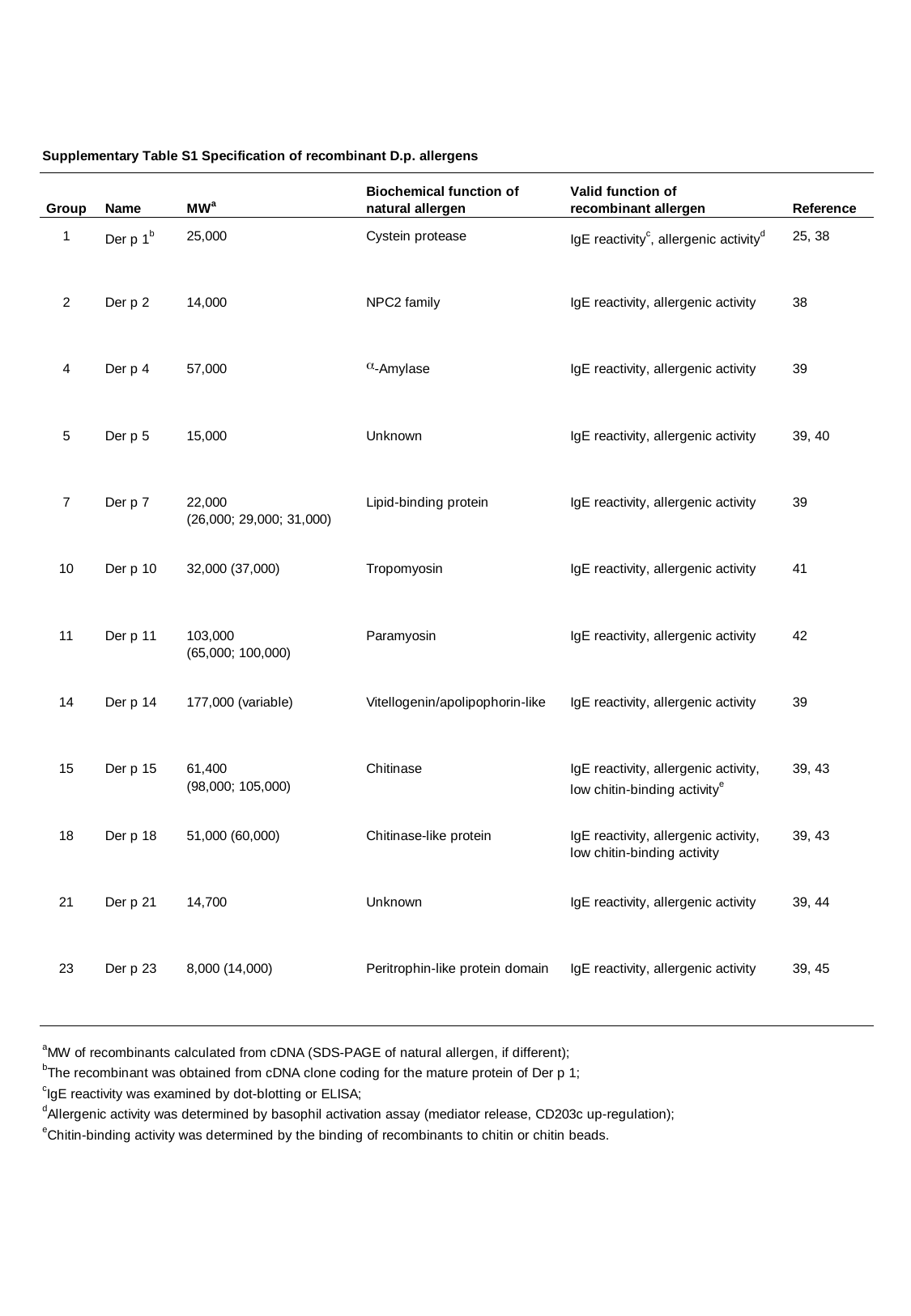

## Slide 9
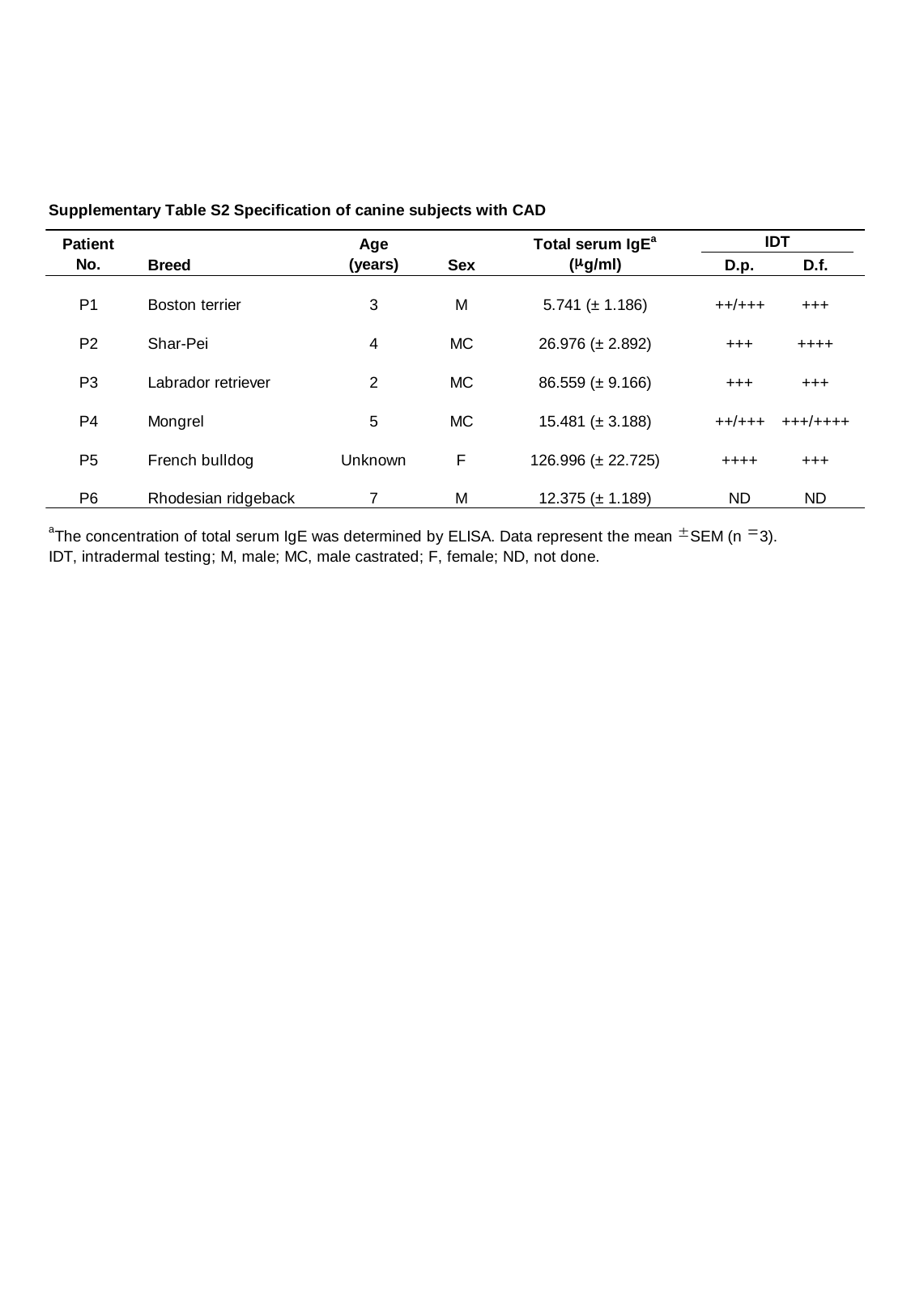

## Slide 10
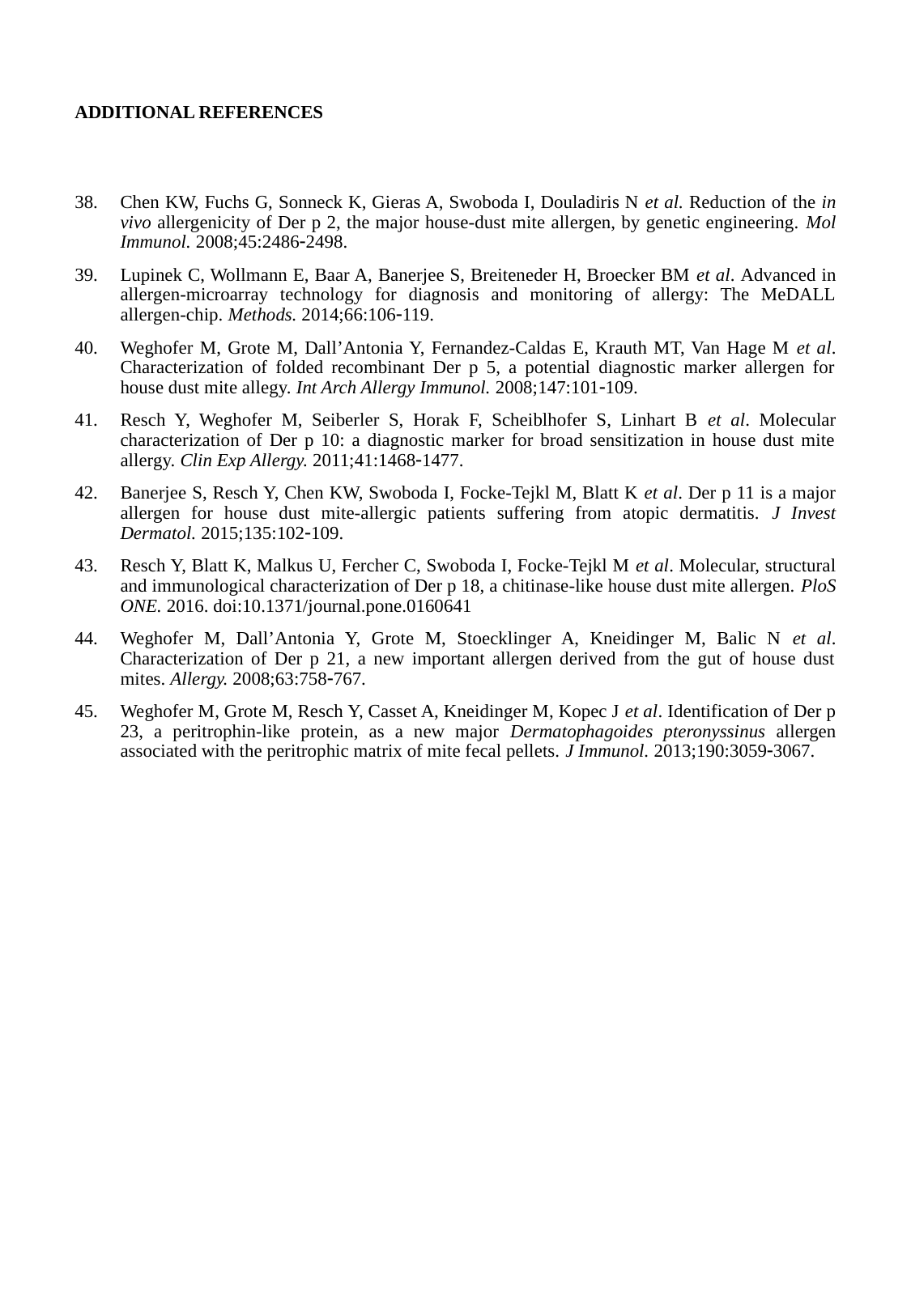

ADDITIONAL REFERENCES
Chen KW, Fuchs G, Sonneck K, Gieras A, Swoboda I, Douladiris N et al. Reduction of the in vivo allergenicity of Der p 2, the major house-dust mite allergen, by genetic engineering. Mol Immunol. 2008;45:2486−2498.
Lupinek C, Wollmann E, Baar A, Banerjee S, Breiteneder H, Broecker BM et al. Advanced in allergen-microarray technology for diagnosis and monitoring of allergy: The MeDALL allergen-chip. Methods. 2014;66:106−119.
Weghofer M, Grote M, Dall’Antonia Y, Fernandez-Caldas E, Krauth MT, Van Hage M et al. Characterization of folded recombinant Der p 5, a potential diagnostic marker allergen for house dust mite allegy. Int Arch Allergy Immunol. 2008;147:101−109.
Resch Y, Weghofer M, Seiberler S, Horak F, Scheiblhofer S, Linhart B et al. Molecular characterization of Der p 10: a diagnostic marker for broad sensitization in house dust mite allergy. Clin Exp Allergy. 2011;41:1468−1477.
Banerjee S, Resch Y, Chen KW, Swoboda I, Focke-Tejkl M, Blatt K et al. Der p 11 is a major allergen for house dust mite-allergic patients suffering from atopic dermatitis. J Invest Dermatol. 2015;135:102−109.
Resch Y, Blatt K, Malkus U, Fercher C, Swoboda I, Focke-Tejkl M et al. Molecular, structural and immunological characterization of Der p 18, a chitinase-like house dust mite allergen. PloS ONE. 2016. doi:10.1371/journal.pone.0160641
Weghofer M, Dall’Antonia Y, Grote M, Stoecklinger A, Kneidinger M, Balic N et al. Characterization of Der p 21, a new important allergen derived from the gut of house dust mites. Allergy. 2008;63:758−767.
Weghofer M, Grote M, Resch Y, Casset A, Kneidinger M, Kopec J et al. Identification of Der p 23, a peritrophin-like protein, as a new major Dermatophagoides pteronyssinus allergen associated with the peritrophic matrix of mite fecal pellets. J Immunol. 2013;190:3059−3067.
